# Identification of fractures in tight-oil reservoirs: a case study of the Da'anzhai member in the central Sichuan Basin, SW China

**DOI:** 10.1038/s41598-021-03297-6

**Published:** 2021-12-13

**Authors:** Jie Tian, Hongqi Liu, Liang Wang, Liqiang Sima, Shiqiong Liu, Xiangjun Liu

**Affiliations:** 1 School of Geosciences and Technology, Southwest Petroleum University, Chengdu, 610500 Sichuan China; 2 College of Earth Sciences, Chengdu University of Technology, Chengdu, 610500 Sichuan China

**Keywords:** Geophysics, Core processes

## Abstract

The Da'anzhai Member of the Jurassic Ziliujing formation in central Sichuan is a typical tight-oil reservoir with porosity and permeability less than 2% and 0.1 × 10^–3^ μm^2^, respectively. Fractures in this formation are well developed in micro- and nano-scale. However, the factors that control the fracture distribution are unclear. Additionally, the uncomprehensive and ineffective identification and evaluation of fractures in the early stage of tight-oil development makes it difficult to meet the requirements of tight-oil development. In our work, we used cores, thin sections, and a scanning electron microscope (SEM) to study the influence of the microscopic rock composition, including the shelly grains, calcite grains, and clastic grains, on the fracture development. We found that the microscopic composition of shelly grains and calcite grains separately control the development of inter-shelly fractures and shelly fractures, and intergranular fractures, and tectonic fractures. Except for a small number of dissolution fractures found in mudstone, the fractures are not well developed in the formations with clastic grains. According to the characteristics of the development degree of fracture and the resolution of the well-logs, the fractures are divided into large scale, small scale, and micro-scale. By a newly established level-by-level constraints method, we systematically identified the scale, occurrence, filling characteristics, and development degree of fractures in the Da'anzhai member by well-logs. Moreover, a quantitative model is also proposed for identifying the angles and development degree of fractures. The results show that the scale of fractures can be effectively identified by the shapes and values of resistivity logs; the occurrence, development, and filling characteristics of fractures can be semi-quantitatively evaluated by the relative amplitude difference between the matrix resistivity (R_*b*_) and formation resistivity (R_*T*_). The results are consistent with the interpretation results by formation micro-resistivity imaging (FMI) log, which further demonstrates that the level-by-level constraint method by conventional well-logs can be used to systematically and effectively predict the fracture characteristics in tight-oil reservoirs.

## Introduction

Unconventional tight-oil and gas resources play a crucial role in China's energy development^1–4^. The Da'anzhai Member of the Ziliujing formation, a wide range of general oil-bearing characteristics^5^, is the leading oil-producing area of the tight-oil reservoirs in the central Sichuan Basin^6^. In the Da'anzhai Member, the structural fractures and a large number of multi-type micro-nano pores and fractures are well developed^7^. Compared with the typical tight-oil reservoirs of Yanchang Formation in Ordos Basin, the Lucaogou Formation in Junggar Basin^8–13^, Bakken^14^, Eagle Ford^15^ and Barnett^16^, the Da'anzhai Member is quite different in its reservoir characteristics, production performance, and fluid properties. It has well-developed natural fractures, good crude oil properties, high-pressure coefficients, low reservoir porosity, and relatively weak hydrocarbon generation capacity^17^^,^^18^. In the early stage of the tight-oil exploration, the large structural fracture is believed to play the dominant role in the tight-oil exploration, and only these kinds of fractures are identified and evaluated. However, with the promotion of the tight-oil exploration, the unstructured fractures with nano-micro scale are demonstrated to play a key role in the tight-oil exploration. Thus, the structural and nano- and micro-scale fractures need to be systematically evaluated to enhance the exploration of the tight-oil.

Traditionally, fractures have been jointly identified by conventional logs of acoustic slowness(AC), compensated neutron(CNL), compensated density(DEN), dual lateral resistivity^19–22^. FMI as an unconventional log is the most intuitive and useful to identify fracture^23^^,^^24^^,^^25^^,^^26^^,^^27^^,^^28^. For the Da'anzhai member of the central Sichuan, with a history of more than 50 years’ oil exploration and development, the majority of wells in this area are old wells with conventional logs. The FMI is only measured in only a few newly-drilled wells due to its high cost. Thus, the FMI log can not be relied on to identify fractures and it is critical to propose a method to identify the fractures by conventional logs.

Many data processing methods such as wavelet transform, adaptive neuro-fuzzy inference system, fractal dimension, probabilistic decision trees are applied to identify fracture in conventional reservoirs by conventional well-logs^29–33^. However, the above methods are proved to be ineffective to identify the fractures due to the weak sensitiveness of the conventional logs to fractures in tight-oil reservoirs^34^. Moreover, the above methods are aimed at specific geological characteristics and only applicable to conventional reservoirs with good physical properties.

In this paper, we took the tight-oil limestone reservoir of Da'anzhai member in central Sichuan as an example, and established a Level-by-level constraints method based on conventional well-logs for identifying fracture: firstly, based on lithology control, the scale and occurrence of fracture are identified; then, based on fracture scale control, the development degree and filling characteristics of fractures are identified, and the identification accuracy is explored to micro-scale fractures. The purpose of this paper is: (1) to analyze the fracture characteristics and fracture control factors of the tight oil reservoirs; (2) to establish a set of fracture identification methods by conventional well-logs.

## Geological background

**Figure 1:**
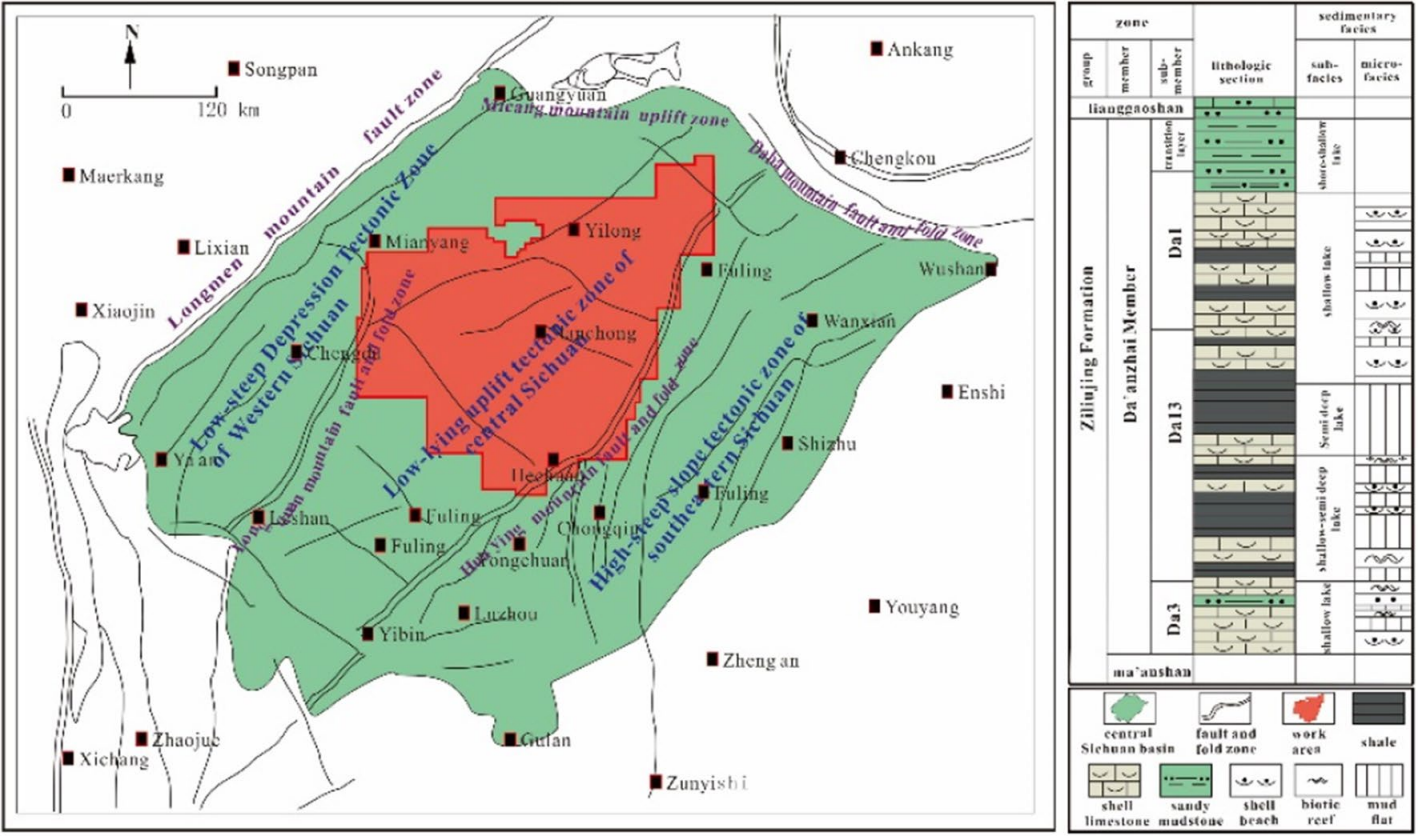
Regional structure map of the central Sichuan (created with CoreDRAW Graphics Suite 2021, https://www.coreldraw.com/en/product/coreldraw/?topNav=en).

The tectonic basin of Sichuan is generally diamond-shaped. It can be divided into three tectonic regions bounded by the anticlines of the Huaying Mountain and the Longquan Mountain: (1) the high-steep slope tectonic zone of the southeastern Sichuan, (2) the low-steep depression tectonic zone of the western Sichuan, and (3) the low-lying uplift tectonic zone of the central Sichuan^35^. The oil and gas region is located in the low-lying uplift tectonic zone of central Sichuan, which is between Longquan Mountain and Huaying Mountain (Fig. 1). Low-amplitude folds dominate the structure of the oil and gas region in various directions and by a few faults^5^. The Da'anzhai member, the main oil-bearing area of the tight-oil reservoirs in central Sichuan, has sedimentary facies typical of continental inland freshwater lacustrine facies deposits. The primary sedimentary environment is a beach facie with shelly grains, and it has a complete longitudinally regression-transgression sedimentary cycle^6^. The lithofacies is mainly interbedded of lacustrine mudstone and shelly limestone with different thicknesses, which have undergone multiple diageneses such as sediment compaction, dissolution cementation, and recrystallization^6^. The average helium porosity and alcohol porosities are 1.77% and 1.04% respectively. The porosity range is 0.26% to 4.31%. The geometric mean of permeability is 0.02 × 10^–3^ μm^2^ with a range of 0.002 μm^2^ to 12.62 × 10^–3^ μm^2^. The characteristics of ultra-low porosity and low permeability demonstrate that the Da'anzhai member is a typical tight-oil reservoir.

In the early stage of the oil exploration and development, the Da'anzhai member was initially considered to be a fracture-type reservoir. Fractures are the reservoir spaces and percolation channels. Thus, the key point for oil exploration was to find fractures with large-scale^7^. With the promotion of oil exploration, both the pores and fractures were found to be oil-bearing, and the Da'anzhai member should not be simply regarded as the fractured-type reservoir. The pores with various types contribute to long-term stable production. Based on the coexistence of the microfractures and matrix pores, the Da'anzhai reservoir was initially defined as a matrix fracture-pore reservoir. In the matrix state, micropores and microfractures are reservoir spaces, and the connections among microfractures provide the channels for the oil migration^36^.

## Basic characteristics of the fractures in the Da'anzhai member

### Characteristics of the fractures

The 178 cores at the production site indicate that the fractures in the Da'anzhai member are mainly structural. The structural fractures in the Da1 sub-member account for 57% of the total fractures, followed by compression-dissolution and interlayer fractures. The Da13 and Da3 sub-members have more than 60% structural fractures, 25% compression-dissolution fractures, and 10% interlayer fractures.

About 80 percents of the fractures are horizontal and low-angle. The Da1, Da13, and Da3 sub-members account for about 55% of the horizontal fractures. The Da1 and Da3 sub-members have about 25% of the low-angle fractures. With 36% high-angle fractures, the high-angle fractures are more common in the Da13 sub-member. The statistics of the 52 cores show that the filling situation of the fractures are 55% closed, 23% semi-filled, and 21% open. The closed fractures and the semi-filled fractures are mainly filled with mud, followed by calcite.

**Figure 2:**
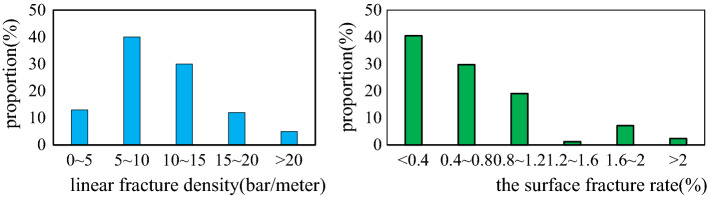
Statistics on the degree of development of different fractures.

The development degree of fractures with large-scale are analyzed by the thin sections and cores. As shown in Fig. 2, the linear density mainly ranges from 5 to 10 fractures per meter. The linear density has a wide range distribution with the maximum linear density approaching 40 fractures per meter. The surface rate of fracture observed from thin sections is mainly in ranges of 0.2–0.6%.

**Figure 3:**
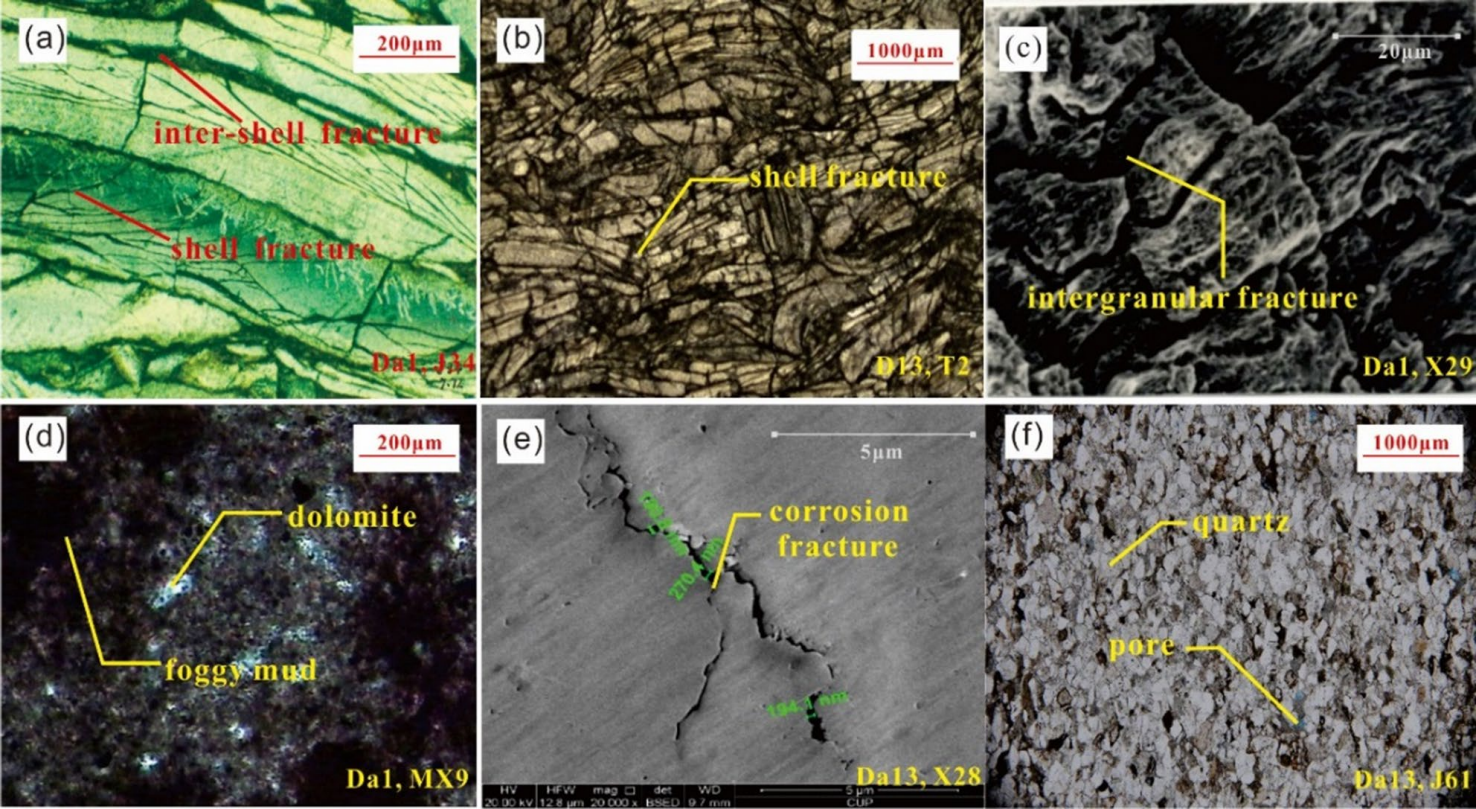
Fracture characteristics of different rock compositions. **(a)** Intershell fractures can be seen along the shells in shell limestone at 2,619.70 m for well J34; **(b)** A large number of connected shell fractures are visible in the clastic limestone at 2,931.06 m for well T1; **(c)** Intergranular fractures developed along the calcite grains, and corrosion traces can be seen in these fractures at 1,991.87 m for well X29; **(d)** Dolomite content is 70%, and no fractures were found at 1,452.0 m for well MX9; **(e)** Corrosion fractures are along the surface of clay minerals, mainly in thin interbed layers in Da13 at 1985.7 m for well X28; **(e)** Intergranular pore was found between quartz grains, but there were no fractures at 2711.42 m for well J61. **(a,b)** are shell-controlled fractures, **(c)** shows matrix-controlled fractures, **(e)** are non-fabric controlled fractures, and **(d,f)** have no fractures.

The characteristics of fracture development in the Da'anzhai member are controlled by the composition and structure of the rocks^7^. The primary organisms contributing to the formation consist of bivalves, gastropods, ostracods, and other shelly microorganisms. A small number of internal clastic particles are included, such as sand and rock debris. The main rock types are shelly limestone, clastic limestone, grain limestone, mudstone, and sandstone. The images of the thin sections and scanning electron microscopy show that (Fig. 3):

- (1) For the shelly limestone with shell contents over 50%, the inter-shell fractures are often developed along the edge of the shells. The shell size and numbers greatly affect the numbers and scales of fractures. The shelly limestone is tight with mainly inter-shell fractures and shell fractures developed. The development and orientation of the inter-shell fractures affect the seepage capacity of the oil;
- (2) For the shelly-clastic limestone, the split of the shell inevitably results in the connection of the inter-shell fractures. The split degree of the shell affects the development of the micro-nano scale fractures;
- (3) For the grain limestone with high calcite content, the types of the minor mesh-intergranular fractures primary develop. The width of these kinds of fractures ranges from 300 nm to 1000 nm. The grain limestone has a strong rock brittleness. It is easy to form structural fractures as to the intergranular fractures, along the weak surfaces under the crustal stress. The size of the crystal grains will affect the scale of the intergranular fractures.
- (4) For the mudstone and sandstone, the primary and secondary intergranular pores develop, and few fractures are observed. The dolomite content in the Da'anzhai member is generally low. The dolomitization process only enlarge the intergranular pore boundary and enhance the pore connectivity, whereas it does not have an obvious influence on the fractures.

### Classification of the fractures

The Da'anzhai member is in the middle-deep diagenetic stage with a medium-deep burial environment. In the early diagenetic stage, strong compaction makes the loss of the primary porosity. There are clay mineral metasomatic particles, calcite, and a small amount of siliceous cementation. During the middle-deep burial diagenesis stage, the dissolution and compressional dissolution is intense. The edges of large structural fractures are dissolved, and the compression-dissolving fractures form in the limestone or at the boundary of limestone and mud^6^. Considering the impact of different scale fractures on hydrocarbon migration and conventional logging resolution, the Da'anzhai member's fractures are classified as large-scale, small-scale, and micro-scale fractures.

As shown in Table 1, the large-scale fractures are visible in the cores, and they are primarily non-fabric controlled fractures. The number of large-scale fractures is small. The small-scale fractures are visible in the thin sections. This kind of fracture includes the structural and dissolution fractures that run through the thin sections and inter-shell fractures with large openings. These fractures are more plentiful than large-scale fractures and are essential for connecting large-scale and micro-scale fractures. The micro-scale fractures have poor connectivity. Scanning electron microscopy shows that irregular and discontinuous fractures appear on the edges of the calcite crystals and shells. The fractures are mostly shell-controlled and matrix-controlled. When the micro-scale fractures are well developed, they interlace and play an essential role in matrix permeability.

**Table 1:** Classification of fractures by scale.

Scale classification	Seam width/mm	Species
Large scale fractures	> 1	Structural fractures, interlaminar fractures, pressure-solution fractures, suture, dissolution enlarged fractures
Small scale fractures	1–0.001	Structural fractures, pressure-solution fractures, dissolution fractures, inter-shell fractures
Micro scale fractures	< 0.001	Shell fractures, fractures in the shell, cleavage fractures, intergranular fractures and dissolution trench

Overall, the micro-scale and small-scale fractures in the Da'anzhai member are highly developed. The oil from the source rocks, such as mud shale, enters the small-scale fractures from the large-scale fractures and then enters the micro-scale fractures. The fractures at different scales are effective channels for oil percolation.

### Level-by-level constraints in fracture identification

For the Da'anzhai member, the conventional well-logs mainly contain AC and dual laterologs, and the CNL and DEN logs are always unavailable. It is challenging to identify fractures systematically by using conventional well-logs. Thus, the level-by-level constraint method is proposed to identify the fracture scale, occurrence, filling characteristics, and development degree.

#### First-level constraints (the exclusion of non-fracture factors)

**Figure 4:**
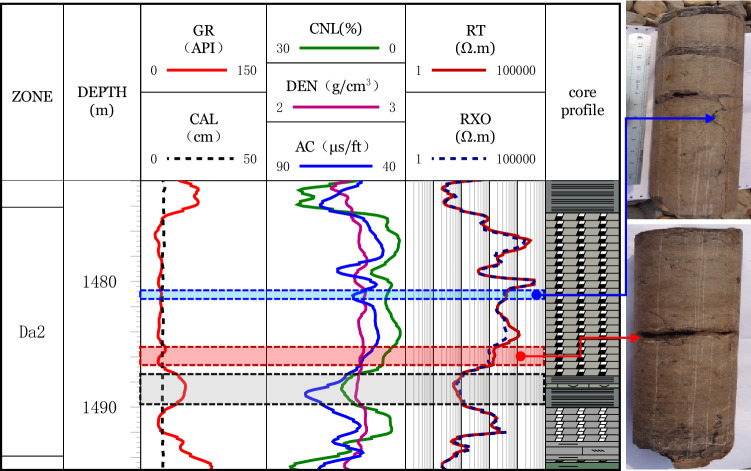
Exclusion of non-fracture log response in layers such as mud bands.

- (1) *Exclusion of thin layers*: Different logging instruments have different resolutions for measuring the thickness of a rock layer. The well-log only reflects the real formation information when the layer thickness is greater than the instrument's resolution. Generally speaking, the resolution of the gamma-ray log(GR) is about 30 cm and the resolutions of AC are 60 cm for limestone and 100 cm for mudstone. The resolutions of CNL and dual laterologs are approximately 40 cm and 80 cm, respectively. Considering the well-logs’ resolutions, a rock layer with a thickness greater than 1 m was selected for fracture identification. The logging values of the thin interbedded layers of the Da13 sub-member are affected by the surrounding rock, so they cannot represent the actual response characteristics of the rock layer.
- (2) *Exclusion of mud impact*: For mud bands, its response is similar to large-scale fractures with high values of AC and CNL and low values of formation resistivity(R_*T*_) and flushed zone resistivity(R_*XO*_). These mud bands can be excluded using a combination of GR and AC. For the mud-filled, the resolution of GR is commonly much larger than the fracture width. Thus, GR has no obvious response to the mud-filled fractures, that is the mud-filled fractures have a low value of GR. The well-logs are characterized by a smooth box or micro-dentation box type shape. However, the values of GR for the mud bands increase significantly and AC has a dentation-finger shape.
- The above characteristic as to the mud bands and mud-filled fractures are shown in well HC125-17-H (Fig. 4). At the depth of the 1481 m to 1483 m, the fractures are well-developed. These fractures are mud-filled as displayed in the core. The lithology is fine-grained limestone. The well-log shows a low value of GR with approximately 23 API. Correspondingly, the values of AC and CNL increase significantly, while the R_*T*_ values decrease significantly. A dissolution fracture at the depth of 1486 m shows a similar logging response to the mud-filled fracture. The logging response of the dissolution fracture has the characteristics of the low GR and RT, and high AC, while no obvious DEN changes. At the depth of 1488–1490 m, a typical mud band develops. Compare to the low GR values of the mud-filled fracture and dissolution fracture, the GR value for the mud band is high. Except for the different GR characteristics, the mud band has similar characteristics of AC, CNL, and R_*T*_ to mud-filled fracture and dissolution fracture.
- (3) *Influence of wellbore stability*: During the drilling process, wellbore instability may occur due to factors such as geological structure, stratum in-situ stress, and weak structural planes. For the intervals with borehole expansion, AC, DEN, and CNL values will be affected. The values of the CNL and AC will increase, while the DEN values will decrease. When the borehole expands dramatically, the values of R_*T*_ and R_*XO*_ will also decrease, indicating a similar well-log response to fractures. The influence caused by the borehole expansion can be excluded by using the caliper log (CAL).

#### Secondary constraints (lithologic constraints)

**Figure 5:**
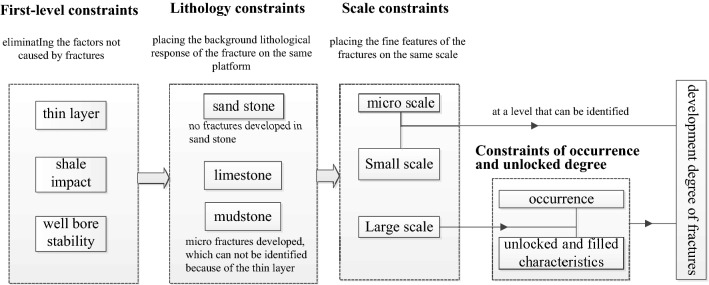
Technology roadmap of level-by-level constraints for fracture identification.

- (1) Lithology constraints on fractures
- After considering the first-level constraints, lithological constraints are imposed on the analysis of fracture development. First, it is necessary to identify the lithologic characteristics of the intervals where fractures are found. The lithology is the background of the log response and the most fundamental factor for controlling the development of fractures. The main lithology of the Da'anzhai member is limestone, mudstone, sandstone, and a small amount of dolomite.
- For the sandstone, calcareous sandstone, and the sandy limestone filled with quartz in the Da'anzhai member, intergranular pores are mainly found among quartz grains, clay minerals, and calcite. The foamy intragranular pores are found within quartz grains. The fractures are not developed and are rarely found in the sandstone.
- For the mudstone in the Da'anzhai member, the reservoir space has intragranular and intergranular pores in clay minerals. The pore size is in the range of 0.1 μm to 1 μm. The pore morphology is irregular, which is related to the clay minerals and hard particles. The development degree of fracture is low, which is mainly found in the Da13 sub-member. The primary type of fractures in the mudstone are pressure-solution fractures, formed by differential compaction and chemical solution pressure among components. Because of the distortion of the well-log and the low width of the pressure-solution fractures, the pressure-solution fractures in the mudstone cannot be identified.
- According to the analysis above, we mainly identified fractures in limestone and dolomite in the Da'anzhai member. Since the dolomite only occupies a small amount of the Da'anzhai members, we focus on the fracture identification in limestone.
- (2) Characteristics of primary lithology response in fracture zones
- From the lithology analysis of the Da'anzhai member, it can be seen that the limestone in the Da'anzhai member is widely distributed, and its structure is mainly composed of calcite crystals and shells, including shells from bivalves and gastropods. The original material is carbonate minerals, including aragonite and calcite. In the process of burial diagenesis, the original aragonite and high-magnesium calcite are converted into low-magnesium calcite. The lithology becomes more homogeneous, and the shale content decreases, which in general, leads to high values of the RT. The well-development of micro-nano-scale reservoir spaces leads to porosity and permeability less than 2% and 0.1 × 10^–3^ μm^2^, respectively. The ultra-low porosity and low permeability lead to the high RT value and low AC value.
- In general, due to the high calcite, low shale contents, and ultra-low porosity and permeability, the RT value of limestone is generally greater than 5000 Ω∙m, the AC values are approaching 48 μs/ft, the GR values are low. For the thick limestone layer, the conventional well-logs have a box-shaped form with smooth or micro-dentation change.
- Figure 5 shows that the scale and origin of fractures can be judged after applying the lithology constraints. The types of fractures that can be identified need to be determined, and they need to be classified as large, small, or micro scale. After this, within the constraints of large-scale fractures, the fracture filling and fracture occurrence are identified. Finally, the development degree of the micro-scale and large-scale fractures is determined. The purpose of level-by-level constraints is to exclude the influence of the non-fracture features on the logging curve and to locate the fracture characteristics to be investigated at a comparable level.

### Identification of fracture characteristics

#### Scale identification of fractures

**Figure 6:**
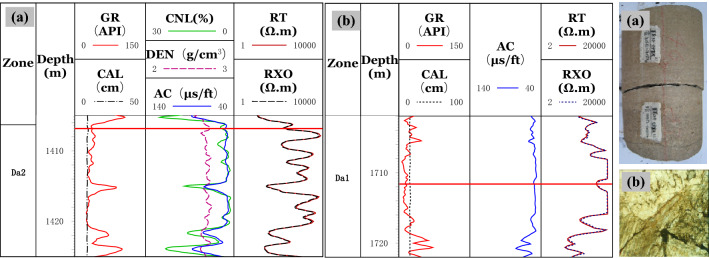
Logging response characteristics of large and small scale fractures. **(a)** A horizontal unlocked-fracture can be seen from the core, and RT values decreased with the RT curve having a dentate shape at 1406.7 m in well PL15; **(b)** A structural fracture runs through a thin section, and the RT values decreased with the RT curve exhibiting a platform notch type at 1411.9 m in well X5.

**Figure 7:**
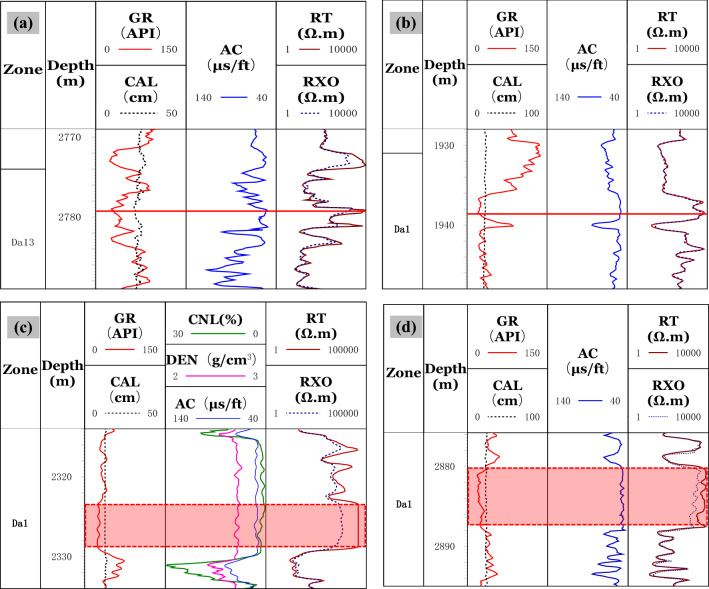
Logging response characteristics of microscale fractures. **(a)** Micro-nano inter-shell fractures develop, and the RT curve has a finger shape at high amplitude at 2778.8 m in well J26; **(b)** micro-nano inter-granular fractures and shell fractures develop, and the RT curve has finger shape at medium amplitude at 1938.4 m in well L21; **(c)** the lithology is tight, and inter shell fractures, shell fractures, and intergranular fractures develop. The calcite content ranges from 98 to 100%, and the RT and RXO curves have a platform double-track shape at 2324 m to 2328 m in well LG83; **(d)** microfractures develop, and the RT and RXO curves have a platform double-track shape at 2881 m to 2887 m in well T2.

**Figure 8:**
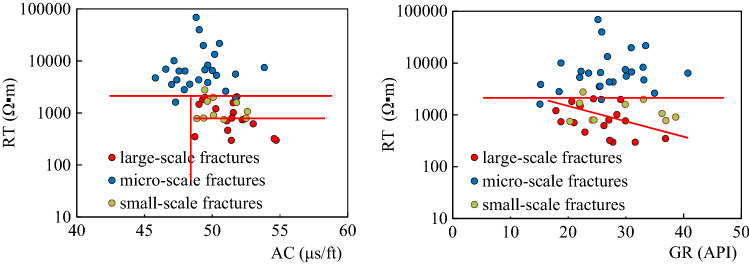
Cross-plot for analysis of fracture scale.

- (1) Logging response characteristics
- The interlaminar fractures, which cannot be effectively identified due to the limited resolution of conventional well-logs, are formed in the thin lime-mud interbeds of the Da13 sub-member. For large-scale structural fractures and dissolution fractures, the mud invasion into the opened fractures will result in a relative decrease of resistivity. The decrease in resistivity is related to fracture width, filling, and radial extension. The R_*T*_ values for the large-scale fractures decrease dramatically, having a dentate shape or a finger shape at the background of high resistivity values. The reduced resistivity is lower than 3,000 Ω▪m. The AC value has an increasing trend with values even higher than 48 μs/ft.
- Compared with large-scale fractures, the width of the small-scale fractures is small, resulting in the high background value of the R_*T*_. The AC values increase slightly. The increasing rate of the AC of the small-scale fractures is lower than that of the large-scale fractures. The R_*T*_ values are about 6,000 Ω▪m, with the R_*T*_ curve exhibiting a dentate drop compared to the matrix rock background, which often drops to a gap shape, and the R_*T*_ curve becomes a "platform notch type", as shown in Fig. 6.
- In limestone, the main types of micro-scale fractures are intergranular fractures and interlaminar fractures. Similar to pores, micro-fractures are spaces in reservoirs with poor connectivity that are mainly found in pure grain limestone and shell limestone with little shale content. Through calibration of a large number of core and thin sections to well-logs, the micro-scale fractures are characterized by low values of GR and AC. The R_*T*_ values are equal to or close to the resistivity of matrix rock. The R_*T*_ log shows "finger-type" and "platform double-track type" shapes.
- *Finger type:* In thick limestone, the R_*T*_ curves for micro-scale fractures show a finger peak, and the R_*T*_ values are equal to or close to the R_*T*_ values of the matrix rock. Due to the difference in lithology and thickness, the R_*T*_ values often have high, medium, and low amplitudes. The shape of the GR curve is smooth box-type with GR value increasing slightly. The AC curve is smooth and box-shaped. Compared with adjacent rock strata with good connectivity or high mud content, the R_*T*_ curve is "finger-type" because of the poor connectivity of the micro-fractures, as shown in Fig. 7a,b.
- *Platform double-track type*: Double rails appear in the lateral resistivity, with a smooth box-shaped curve and a high resistivity value of 6,000 Ω▪m. Micro-fractures are formed by stress release in dense intervals. The general shape of the GR and AC curves is box-shaped. The curves have micro-dentation with values increasing slightly, as shown in Fig. 7c,d. The double-rail phenomenon of micro-scale fractures is caused by the deformed high-angle fractures along weak structural planes. At the same time, due to the widespread oil-bearing in the Da'anzhai member, the mud invasion in a limestone formation has a low resistivity, forming a "platform double rails" phenomenon for the micro-scale fractures.
- (2) Cross-plot identification
- Within the constraints of layer thickness, mud content, caliper precision, and lithology, the GR, R_*T*_, and AC values of fractures with different scales are determined. In thick limestone layers, the GR values are in the range of 10 API to 40 API. Micro-scale fractures have higher R_*T*_ values and lower AC values than small-scale and large-scale fractures. The R_*T*_ values are often over 6000 Ω·m with AC values within the range of 46 μs/ft to 50 μs/ft. On the contrary, the R_*T*_ values for the large-scale fractures are lower than those of the small-scale and micro-scale fractures with values as low as 200 Ω·m. The AC values of the large-scale fractures can reach up to 55 μs/ft as shown in Fig. 8.

#### Identification of fracture occurrence

**Figure 9:**
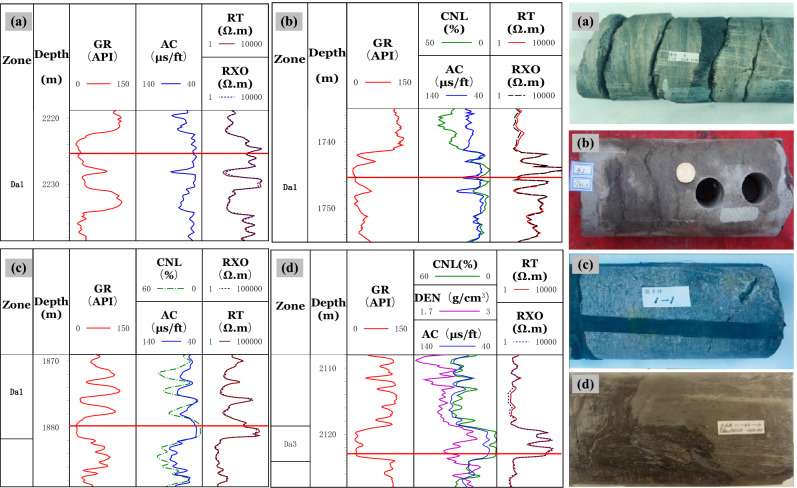
Logging response characteristics of different occurrences of fractures. **(a)** Logging response for low angle fractures show that five low-angle structural fractures are nearly parallel, and the RT values decrease significantly from 4337 to 500 Ω·m (Well L16 at 2225.6 m). **(b)** Three horizontal fractures can be seen from the core, partly filled with mud. The RT values decrease from 7000 to 50 Ω·m, and the RT curve has a spike-like shape. The DEN and the AC values clearly increase (Well X8 at 1745.6 m). **(c)** Oblique opened fractures can be seen in the core. The AC and the GR values have no obvious changes. The RT values decrease to about 1500 Ω·m, and the RT curve has in a dentate shape (Well C2 at 1879 m). **(d)** High-angle fractures can be seen from the core. The GR, AC, and RT values are the same as those of the matrix rock section (Well W6 at 2122.98 m).

**Figure 10:**
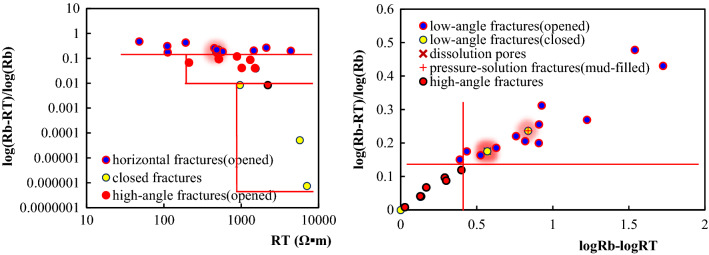
Cross-plot identification of fracture occurrence.

- (1) Logging response characteristics
- Within the constraints on large-scale fractures, an analysis of logging response characteristics shows that the response for high-angle and oblique fractures is unclear. In contrast, the response for low-angle fractures and horizontal fractures is obvious. AC and R_*T*_ values decreased, and their curves are finger-shaped.
- Figure 9 shows that the GR log curve is box-shaped and smooth for low angle and horizontal fractures. The R_*T*_ curve is often reduced to a spike-like or dentate shape. There is either no amplitude difference or a slight negative amplitude difference. The AC values tend to increase, yielding an AC curve with a spike-like or dentate shape. According to their logging curves, the RT values are lower for the oblique fractures, and the AC values are slightly higher. For high-angle fractures in the Da'anzhai member, due to their low development degree, intense filling, and small opening, the well-logs show matrix rock characteristics.
- (2) Cross-plot identification
- Within the constraints of large-scale fractures, we analyzed the cross-plot of the fracture angles. Due to the few data points of the low-angle fractures and the similar logging response of horizontal fractures to low-angle fractures, we combine and treat the low-angle as horizontal fractures when cross-plot analyzing.
- The AC values of high angle fractures are in the range of 40 μs/ft to 60 μs/ft, and the R_*T*_ values are greater than 800 Ω·m. Compared with high angle fractures, the AC values of low angle fractures are in the range of 40 μs/ft to 80 μs/ft. The R_*T*_ values can be as low as 54 Ω∙m.
- As the horizontal fractures have a finger-like decline on the R_*T*_ log, high-angle fractures have no obvious response. The fracture occurrence can be distinguished by using the relative difference between logarithmic matrix rock resistivity (logR_*b*_) and logarithmic formation resistivity (log R_*T*_ ). On the cross plot of (logR_*b*_-logR_*T*_)/logR_*b*_ versus (logR_*b*_-logR_*T*_), the high-angle fractures, and the horizontal open fractures form a distinct partition. The relative difference of the matrix rock resistivity for the horizontal fractures is larger, (logR_*b*_-logR_*T*_)/logR_*b*_ > 0.15. The relative difference between logR_*T*_ and logR_*b*_ for horizontal closed fractures is close to 0.
- It should be pointed out that (logR_*b*_-logR_*T*_)/logR_*b*_ increases when the horizontal fracture opens is filled with mud or when the corresponding cores develop dissolution cavities, and the data points will be distributed in the horizontal fracture range. The (logR_*b*_-logR_*T*_)/logR_*b*_ versus R_*T*_ cross plot shows the differences among horizontal fractures, high-angle fractures, and closed fractures, as shown in Fig. 10.

#### Identification of fracture filling conditions

**Figure 11:**
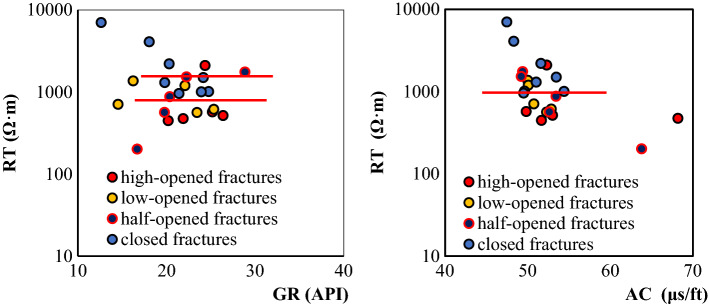
Cross-plot for the opening degree for large-scale fractures.

**Figure 12:**
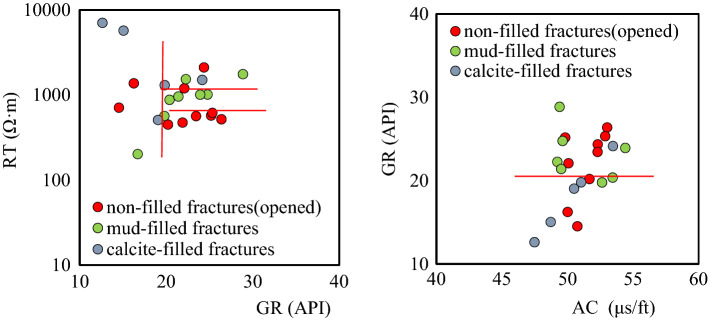
Cross-plot for the filling types of large-scale fractures.

- (1) Degree of fracture opening
- We found that the R_*T*_ values of closed fractures are greater than 800 Ω·m, and (logR_*b*_-logR_*T*_)/logR_*b*_ is less than 0.1. The opening degree of fractures is classified as a high-degree opening, low-degree opening, half-open, and closed. As shown in Fig. 11, only the R_*T*_ values have obvious differences for fractures with different opening degrees. The R_*T*_ values of closed and half-open fractures are higher than those of open fractures. With the same GR background value, the R_*T*_ values of closed fractures are higher than those of half-open fractures. The higher the opening degree, the lower the R_*T*_ values are.
- (2) Fracture filling situation
- The GR values of the mud-filled fractures are significantly higher than those of the calcite-filled fractures, and the boundary line is 20 API. The AC values of the mud-filled fractures can reach 63 μs/ft, which is equivalent to that of open fractures, so AC values are not good indicators of fracture filling. The R_*T*_ values of the calcite-filled fractures are significantly larger than those of the mud-filled fractures, and the R_*T*_ values of the mud-filled fractures are higher than those of the non-filled open fractures. The GR values of the non-filled fractures are slightly higher than those of the filled fractures, and their R_*T*_ values are low. The specific classification criteria are shown in Fig. 12 and Table 2.

**Table 2:** Identification criteria for large-scale fracture filling.

Large-scale fractures	GR (API)	AC (μs/ft)	R_*T*_ (Ω·m)
Non-filled	10–30	50–55	800–2000
Mud-filled	> 20	48–52	100–2000
Calcite-filled	< 21	49–65	1000–10,000

#### Identification of the development degree of fractures

**Figure 13:**
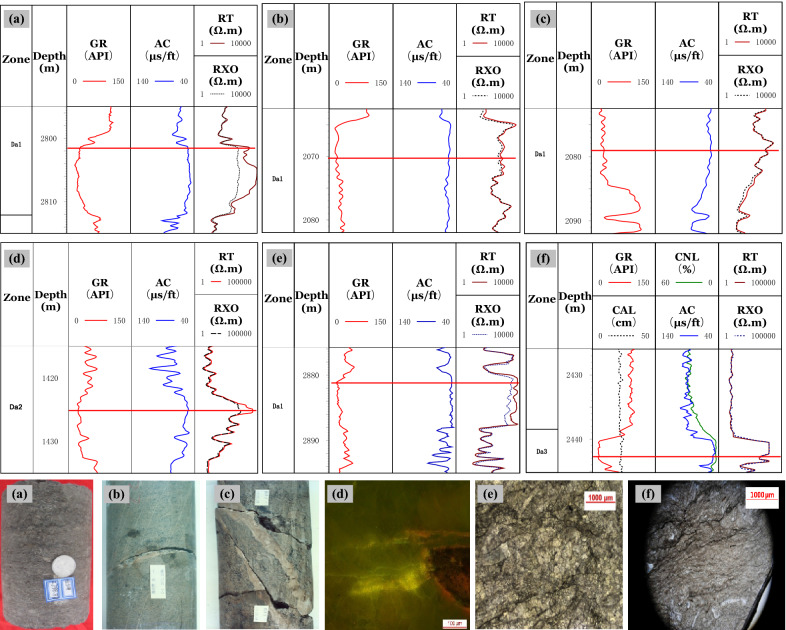
Logging response maps for different scales at different degrees of development. **(a)** The linear density of fractures is low. The RT values are medium–high at 3400 Ω·m, and the RT curve has in a dentate shape (Well T5 at 2801.5 m). **(b)** The linear density of fractures is medium. The RT values are medium–high (Well X46 at 2070.4 m). **(c)** High linear density of fractures can be seen from the core. The RT values decrease to 1400 Ω·m, and RT curve has a dentate shape (Well X46 at 2878.8 m). **(d)** The surface fracture rate is 0.5%. The RT values are extremely high at 36,080 Ω·m, and the RT curve has a fingertip shape (Well M030 at 1425.2 m). **(e)** The surface fracture rate is 1%, and the RT values are high at 7001 Ω·m (Well T2 at 2881.2 m). **(f)** The surface fracture rate is 2.25%, and several structural fractures can be seen from the thin section. The RT curve has a “platform notch type” shape, and the RT value is 3800 Ω·m (Well G4 at 2442.9 m).

**Figure 14:**
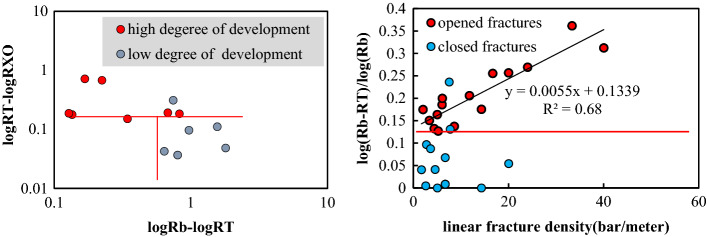
Interpretation chart of fracture development.

- (1) logging response characteristics
- The development degree of fracture, especially the open fractures will affect the logging response reservoir effectiveness. Therefore, in the process of quantitative identification of the development degree of fracture, only the development degree of the open fractures at different scales are studied. On the whole, whether it is a large-scale fracture, a small-scale, or a micro-scale fracture, the higher the development degree of a fracture is, the more distinct the decrease of the resistivity is. The AC values will increase with an increasing development degree of fractures.
- Figure 13 shows that for large-scale fractures, increasing linear density leads to increasing AC values and decreasing R_*T*_ values, which can be reduced to several hundred Ω∙m. Surface fracture rates and well-logs can be used to quantitatively analyze the development degree of micro-scale and small-scale fractures.
- In general, the development degree of micro-and small-scale fractures has no obvious effect on GR and AC curves, while an obvious effect on the R_*T*_ . Increasing surface fracture rates lead to decreasing absolute values of R_*T*_. The R_*T*_ values of micro-and small-scale fractures are lower than that of matrix rock, and their R_*T*_ log has a dentate shape.
- (2) Quantitative identification of the developmental degree
- Based on the different characteristics of resistivity of large-scale and small-scale fractures with different development degrees, we selected the difference between the R_*b*_ and the R_*T*_ values to analyze the linear density of large-scale fractures. Moreover, we selected the difference of amplitude between the R_*T*_ and R_*XO*_ values to identify the development degree of small and micro-scale fractures. Due to the low resolution of the well-logs, it was difficult to identify the surface fracture rate of micro-scale and small-scale fractures quantitatively by conventional logging curves.
- The higher the difference between the R_*T*_ and R_*XO*_ values, the higher the development degrees of the micro-and small-scale fractures. The boundary value between the high and the low development of fractures for logR_*T*_-logR_*XO*_ is 0.1. For the development of large-scale fractures, we found that the linear density has a good positive correlation with (logR_*b*_-logR_*T*_)/logR_*b*_. The higher the linear density, the greater the reduction of the R_*T*_ values is and approach to the matrix resistivity. For the same AC, the higher the linear density of fractures is, the lower the R_*T*_ value is. The specific identification criteria are shown in Fig. 14 and Table 3.

**Table 3:** Interpretation criteria for fracture development.

Types	Fine class	(logR_b_ − logR_T_)/logR_b_	logR_T_ − logR_XO_	GR	AC
Microscale	High developed	0.1–0.2	> 0.1	16–42	45–60
	Low developed	0.1–0.2	0.01–0.1	16–42	45–60
Large scale	Open fractures	> 0.1	149.46 × ((logR_b_-logR_T_)/logR_b_) − 17.587		

## Discussion

**Figure 15:**
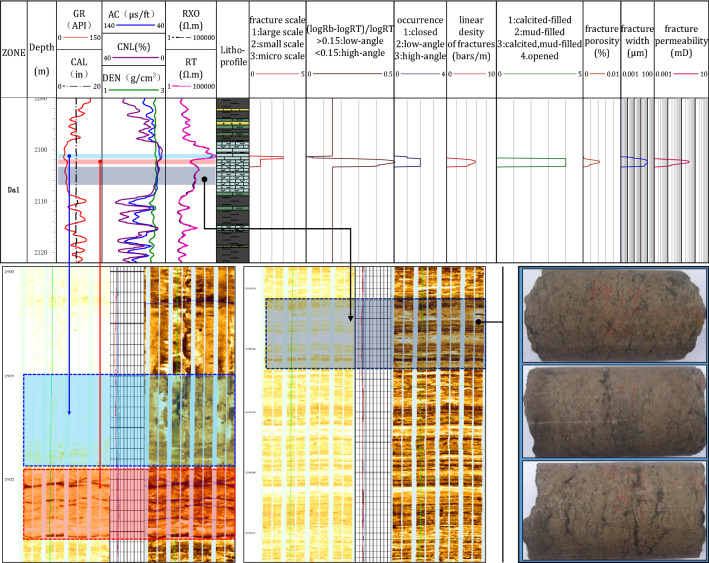
Comparison of fracture parameter interpretation effects.

Based on the identification criteria for fracture scales and fillings and the models of fracture angles, development degree, openness, porosity, and permeability (Table 4), the fracture parameters can be calculated. Taking the well LQ2 as an example, the interpretation correctness and the applicability of the level-by-level constraint method is discussed as shown in Fig. 15.

**Table 4:** The models of fracture interpretation.

Model type	Formula	Parameter description
Porosity	${\Phi }_{f}=\sqrt[mf]{{R}_{m}(\frac{1}{{K}_{r}{R}_{xo}}-\frac{1}{{R}_{T}})}$	Φ_*f*_——fracture porosity, % R*m*——mud filtrate resistivity, Ω∙m R_*XO*_——shallow lateral resistivity, Ω∙m R_*T*_——deep lateral resistivity, Ω∙m K*r*——the coefficient of fracture distortion, in the range of 1–1.3. Itis 1.3 for horizontal fractures and 1 for vertical fractures m_*f*_——fracture bond index, constant ε—— fracture width, μm R_*b*_——matrix rock resistivity, Ω∙m K_*f*_——fracture penetration rate
Fracture angle	logR_*b*_ − logR_*T*_/logR_*b*_(> 0.15, high-angle fractures; 0.05 ~ 0.15, low-angle fractures)	
High-angle unlocked fracture	ε = 10,000 × Rm × ($\frac{1}{{R}_{T}}-\frac{1}{{R}_{b}}$)/4	
Low-angle unlocked fracture	ε = 1000 × Rm × ($\frac{1}{{R}_{T}}-\frac{1}{{R}_{b}}$)/1.2	
Permeability	K_*f*_ = 8.50 × 10^–4^ × ε^2^ × Φ_*f*_	

For the value of the fracture cementation index *m*_*f*_, the *m*_*f*_ of the large-scale fractures is 1.04, and the *m*_*f*_ of the structural fractures is 0.99 (derived from the rock data).

At the depth of 2101 m to 2102 m, the resistivity is extremely high, the AC values and the CNL values are low, and the DEN values are high, showing obvious compact lithologic response characteristics for conventional well-logs. By the level-by-level constraint method, we identified micro-scale fractures as the predominant fractures in this layer. The static images of the FMI show high-brightness and compact intervals, while the dynamic images of the FMI show dissolution pores and no large-scale fractures. The interpretations of the conventional well-logs are consistent with the interpretations by FMI. The comparison shows that the level-by-level constraint method of fracture identification is effective.

At 2102 m, the R_*T*_ curve shows a marked decline with a dentate shape, the CNL values show a significant increase, and the AC values also increase. By using the level-by-level constraint method, the fractures are identified as large-scale open fractures with low angles and no filling. The development degree of fracture can be characterized as five fractures per meter, average fracture porosity of 0.002%, and fracture width of 10 μm. Both the static and dynamic FMI images show six horizontal open fractures distributed in black bands. The logging interpretation by conventional well-logs is consistent with the interpretation by FMI. Additionally, the comparison shows that the level-by-level constraint method based on conventional well-logs is suitable for interpreting the fracture intervals.

At the depth of 2102 m to 2108 m, the CNL values increased continuously, and the R_*T*_ values decreased significantly compared to the values from 2101 to 2102 m, but there are similar fracture response characteristics. The increased range of the CNL values and the decreased range of the R_*T*_ values are lower than those of fractures at 2102 m. The average value of GR is higher than that from 2101 to 2102 m. The conventional well-logs reflect an increase in mud contents. During the identification process by the level-by-level constraint method, the fractures can not be identified due to mud contents and lithology constraints. The static FMI shows a high resistivity and dense lithology of fractures from 2012 to 2018 m, and low resistivity bands can be seen under high resistivity. On the dynamic FMI, dark and black bands are parallel, which is obvious muddy bands. The core shows that the lithology is mesoclastic limestone, and an argillaceous strip can be observed.

Overall, the fracture identification by conventional well-logs at different depths in well LQ2 is consistent with FMI. It shows that the level-by-level constraint method can systematically and effectively interpret the fractures of tight-oil reservoirs in the Da'anzhai member of central Sichuan based on conventional well-logs.

## Conclusion

This paper proposes an effective method to evaluate the scale, occurrence, and development degree of the fractures using conventional well-logs. The main conclusions are as follows:

- (1) The composition and structure of the rocks in the Da'anzhai member will affect the development of the fractures. The composition of the shells controls the development of the inter-shell fractures and the shell fractures. The calcite grain composition controls intergranular fractures and the development of tectonic fractures. The fractures in sandstone and mudstone are undeveloped, and only a small number of dissolution fractures are found in mudstone.
- (2) Large-scale fractures of the Da'anzhai member can be identified from both AC and RT logs. The R_*T*_ values of large-scale fractures are lower than 3,000 Ω·m, and the R_*T*_ curves often have dentate or finger-like shapes. The resistivity curves of small-scale fractures have a platform-notch shape with values of about 6,000 Ω·m. The micro-scale fractures have high resistivity values in finger shape. The smaller the scale is, the closer the resistivity is to the resistivity of the matrix value.
- (3) High-angle fractures of the Da'anzhai member are poorly developed and often filled. The high-angle fractures have an unobvious response in the well-logs. The resistivity log for horizontal fractures and low-angle fractures show finger-like decreases, and the AC values increase.
- (4) Increasing the linear density of large-scale fractures leads to a decrease in the dentate character. The RT value decreases with the increasing surface fracture rate for micro-scale fractures.
- (5) The level-by-level constraint method can be used to identify the fractures systematically and effectively in the tight-oil reservoirs of the Da'anzhai member, which is of great significance to the development of tight-oil in old oilfields.

## References

[1] Jia CZ, Zheng M, Zhang YF (2012). Unconventional hydrocarbon resources in China and the prospect of exploration and development. Pet. Explor. Dev..

[2] Zou CN, Yang Z, Hou LH, Zhu RK, Cui JW, Wu ST (2015). Geological characteristics and "sweet area" evaluation for tight oil. Pet. Sci..

[3] Hu SY, Zhu RK, Wu ST, Bai B, Yang Z, Cui JW (2018). Exploration and development of continental tight oil in China. Pet. Explor. Dev..

[4] Guo, Q. L., Wang, S. J., Chen, X. M. Assessment on tight oil resources in major basins in China. *J. Asian Earth Sci.* **178**, 52–63. 10.1016/j.jseaes.2018.04.039 (2019).

[5] Xu QL, Liu B, Ma YS, Song XM, Wang YJ, Chen ZX (2017). Geological and geochemical characterization of lacustrine shale: A case study of the Jurassic Da'anzhai member shale in the central Sichuan Basin, southwest China. J. Nat. Gas Sci. Eng..

[6] Yang G, Huang D, Huang PH, Yan WP, Yang T (2017). Control factors of high and stable production of Jurassic Da'anzhai Member tight oil in central Sichuan Basin, SW China. Petrol. Explor. Dev..

[7] Tian ZP, Song XM, Wang YJ (2017). Classification of lacustrine tight limestone considering matrix pores or fractures: A case study of Da'anzhai Member of Jurassic Ziliujing Formation in central Sichuan Basin, SW China. Petrol. Explor. Dev..

[8] Yao, J. L. *et al*. Characteristics of tight oil in Triassic Yanchang Formation, Ordos Basin. *Petrol. Explor. Dev.* **40**(2), 161–169. 10.1016/s1876-3804(13)60019-1 (2013).

[9] Ju, W. *et al*. Present-day in-situ stress field within the Yanchang Formation tight oil reservoir of Ordos Basin, central China. *J. Petrol. Sci. Eng.* **187** (2020).

[10] Wang, Y. *et al*. The forming mechanism and process of tight oil sand reservoirs: A case study of Chang 8 oil layers of the Upper Triassic Yanchang Formation in the western Jiyuan area of the Ordos Basin, China. *J. Petrol. Sci. Eng*. **158**, 29–46. 10.1016/j.petrol.2017.08.026 (2017).

[11] Kuang LC, Tang Y, Lei DW, Chang QS, Ouyang M, Hou LH, Liu DG (2012). Formation conditions and exploration potential of tight oil in the Permian saline lacustrine dolomitic rock, Junggar Basin, NW China. Petrol. Explor. Dev..

[12] Cao Z, Liu GD, Kong YH, Wang CY, Niu ZC, Zhang JY (2019). Geochemical characteristics of crude oil from a tight oil reservoir in the Lucaogou Formation, Jimusar sag. AAPG Bull..

[13] Li, Z., Liu, Y. Q., Xin, J. *et al*. The characteristics of the hydrothermal exhalative dolostone of Lucaogou Formation in Santanghu basin and its geology setting indication. *Acta Geol. Sin. (English Edition)* **1**, 145. 10.1111/1755-6724.13221 (2017).

[14] Chalmers GR, Bustin RM, Power IM (2012). Characterization of gas shale pore systems by porosimetry, pycnometry, surface area, and field emission scanning electron microscopy/transmission electron microscopy image analyses: Examples from the Barnett, Woodford, Haynesville, Marcellus, and Doig unit. AAPG Bull..

[15] Hsu SC, Nelson PP (2002). Characterization of Eagle Ford Shale. Eng. Geol..

[16] Aydemir A (2011). Comparison of Mississippian Barnett Shale, Northern-Central Texas, USA and Silurian Dadas Formation in Southeast Turkey. J. Petrol. Sci. Eng..

[17] Liu YL, Zhang YZ, Wang YJ, Wang LG (2018). The pore structure of tight limestoneJurassic Ziliujing Formation, Central Sichuan Basin, China. Appl. Geophys..

[18] Yang YM, Yang JJ, Yang G, Tao SZ, Ni C, Lin JP (2016). New research progress of Jurassic tight oil in cental Sichuan basin. Pet. Explor. Dev..

[19] Aguilera, R. Analysis of naturally fractured reservoirs from sonic and resistivity logs. *J. Petrol. Technol.* **26**(11), 1233–1238. 10.2118/4398-PA (1974).

[20] Aguilera R (1976). Analysis of naturally fractured reservoirs from conventional well logs (includes associated papers 6420 and 6421). J. Petrol. Technol..

[21] Nelson R A. *Geologic Analysis of Naturally Fractured Reservoirs*. 2nd edn. 135–149. (Gulf Professional Publishing, 1985).

[22] Sibbit, A. M., & Faivre, O. The dual laterolog response in fractured rocks: Society of Petrophysicists and Well Log Analysts. in *26th Annual Logging Symposium, Dallas,Texas*. 1–34. June 17–20, 1985 (1985).

[23] Yang HJ, Sun SZD, Cai LL, Xiao YJ, Wang HY, Luo CS, Hu HR (2011). A new method of formation evaluation for fractured and caved carbonate reservoirs: A case study from the Lundong area, Tarim Basin, China. Petrol. Sci..

[24] Deng SG, Wang Y, Hu YY, Ge XM, He XQ (2013). Integrated petrophysical log characterization for tight carbonate reservoir effectiveness: A case study from the Longgang area, Sichuan Basin, China. Petrol. Sci..

[25] Zhang YZ, Zeng LB, Luo Q, Zhu RK, Lyu WY, Liu DD (2021). Influence of natural fractures on tight oil migration and production: A case study of Permian Lucaogou Formation in Jimsar Sag, Junggar Basin, NW China. J. Earth Sci..

[26] Yin, S., Tian, T., & Wu, Z. H. Developmental characteristics and distribution law of fractures in a tight sandstone reservoir in a low-amplitude tectonic zone, eastern Ordos Basin, China. *Geol. J.* **55**(2), 1546–1562. 10.1002/gj.3521 (2020).

[27] Wang PW, Jin ZJ, Pang XQ, Guo YC, Chen X, Guan H (2018). Characteristics of dual media in tight-sand gas reservoirs and its impact on reservoir quality: A case study of the Jurassic reservoir from the Kuqa Depression, Tarim Basin, Northwest China. Geol. J..

[28] Wang YB, Yin S, Guo MQ, Zhao JZ, Li J (2020). Fracture logging evaluation of upper paleozoic tight sandstone gas reservoirs in the eastern margin of the ordos basin. Fresenius Environ. Bull..

[29] Zhang XF, Pan BZ, Wang F, Han X (2011). A study of wavelet transforms applied for fracture identification and fracture density evaluation. Appl. Geophys..

[30] Bagaria R, Wadhwani S, Wadhwani AK (2021). A wavelet transform and neural network based segmentation & classification system for bone fracture detection-sciencedirect. Optik.

[31] Ja'fari A, Kadkhodaie-Ilkhchi A, Sharghi Y, Ghanavati K (2012). Fracture density estimation from petrophysical log data using the adaptive neuro-fuzzy inference system. J. Geophys. Eng..

[32] Leal, J. A., Ochoa, L. H., Garcia, J. A. Identification of natural fractures using resistive image logs, fractal dimension and support vector machines. *Ingenieria Invest.* **36**(3), 125–132. 10.15446/ing.investig.v36n3.56198 (2016).

[33] Ozkaya SI (2008). Using probabilistic decision trees to detect fracture corridors from dynamic data in mature oilfields. SPE Reserv. Eval. Eng..

[34] Lyu, W. Y., Zeng, L. B., Liu, Z. Q., Liu, G. P., Zu, K. W. *AAPG Bull.* **100**(09), 1399–1417.10.1306/04041615129 (2016).

[35] Guo, Z. W. *et al*. *Sichuan Basin Formation and Development*. 113–138. (Geological Publishing House, 1996).

[36] Bo Li, Xingzhi W, Hongqi L, Yongjun W, Jie T (2020). Tight carbonate microstructure and its controls: A case study of lower Jurassic da'anzhai member, central Sichuan basin. Acta Geol. Sin. (English edition).

